# Tuberculosis in people with rheumatic disease in Finland 1995–2007: a nationwide retrospective register study

**DOI:** 10.1093/rap/rkz020

**Published:** 2019-08-01

**Authors:** Marjo Vuorela, Nina J Mars, Juha Salonen, Markku J Kauppi

**Affiliations:** 1 Internal Medicine Department, Päijät-Häme Central Hospital, Lahti; 2 Faculty of Medicine, University of Helsinki; 3 Institute for Molecular Medicine Finland (FIMM), HiLIFE, University of Helsinki, Helsinki; 4 Internal Medicine Department, Vaasa Central Hospital, Vaasa, Finland

**Keywords:** rheumatoid arthritis, rheumatic disease, tuberculosis incidence, tumour necrosis factor inhibitors, latent tuberculosis, pulmonary tuberculosis, disseminated tuberculosis, DMARDs

## Abstract

**Objectives:**

RA and its medication, especially TNF-α inhibitors, increase the risk of clinical tuberculosis (TB) infection. We aimed to investigate the clinical manifestations, incidence and temporal changes in TB occurring concurrently with rheumatic diseases (RDs) between 1995 and 2007.

**Methods:**

We combined the register of the Social Insurance Institution of Finland and the National Infectious Disease Register to find adult patients with reimbursed DMARDs and with a TB notification between 1995 and 2007. After reviewing the medical records, we described their clinical manifestations and medications, explored TB incidence trends using Poisson regression, and compared the incidence of TB with that of the general population.

**Results:**

We identified 291 patients with both TB and rheumatic disease (RD), 196 of whom had RA. Between 1995 and 2007, the incidence of TB in adult RD decreased from 58.8 to 30.0 per 100 000 (trend *P* < 0.001, average marginal effect −3.4/100 000 per year, 95% CI −4.4, −2.4). Compared with the general population, the incidence was ∼4-fold. Among RD patients, pulmonary TB was the most common form of TB (72.6%). Disseminated TB was present in 56 (19.6%) patients.

**Conclusion:**

The incidence of TB among RD patients was ∼4-fold that of the general population, and it declined between 1995 and 2007. Disseminated TB was present in nearly 20% of patients.

**Key messages**

- The incidence of tuberculosis among rheumatic disease patients halved between 1995 and 2007 in Finland.
- Compared with that of the general population, the incidence of tuberculosis among rheumatic disease patients was ∼4-fold.
- Disseminated and extra-pulmonary forms of tuberculosis appeared in up to 46.5% of rheumatic disease patients.

## Introduction

In Finland, the annual incidence rate of RA is 45/100 000, and its prevalence in the adult population is 0.8% [1, 2]. RA and its medication, especially TNF-α inhibitors (TNFi), increase the risk of active tuberculosis (TB) infection [3–5]. In Korea, a country with a high incidence of TB, the risk of TB between 2001 and 2005 was 8.9 times as high for patients with RA as for the general population and 30.1 times as high for RA patients treated with infliximab [6]. Reactivation of latent tuberculosis infection (LTBI) during TNFi treatment was first reported in 2001 and is now well documented [7–9]. This risk can be reduced by routine screening and treatment of LTBI at the start of immunosuppressive treatment [9, 10]. The national Finnish guidelines for screening and treating LTBI in RA patients were published in 2004 [11].

Besides RA medication, the increased risk of TB in RA patients may also be related to immunological disturbances associated with the disease, to genetic components, co-morbidities and frailty induced by the disease and medications [12–16]. A study from Sweden (a country with a low incidence of TB) found that compared with the general population, even RA patients not exposed to biologics had a 4-fold increased risk of TB [17]. Owing to the previous high incidence of TB in Finland, those born in Finland before 1950 have had a high likelihood of exposure to TB in childhood [18]. In 2001, Finland became a low-incidence country, with a TB incidence below 10/100 000 [19]. The incidence of TB in RA and rheumatic diseases (RDs) in Finland is as yet unknown.

During TNFi treatment, TB in RA patients is often disseminated or extra-pulmonary [7, 8, 20], but clinical manifestations of TB during treatment with synthetic DMARDs (sDMARDs) or in rheumatic disease (RD) patients without specific medication remain poorly documented.

We created a nationwide retrospective register study, with detailed individual data for patients who developed active TB between 1995 and 2007, to explore the incidence of TB in RA and in other RDs. Between 2001 and 2007, several TNFi became available and gradually became more generally used. We thus sought clinically relevant findings in patients with RD complicated by TB in this population-based series.

## Methods

### Data sources

We combined two nationwide data sources. The register of the Social Insurance Institution of Finland lists people with reimbursed DMARDs since 1966. The national health insurance scheme covers the entire population of Finland, which was 5.28 million in 2007 [21]. Eligibility requires a comprehensive medical certificate written by the attending physician and approved by an expert adviser. All connective-tissue diseases, RA and comparable diseases are grouped under one reimbursement code in the Social Insurance Institution of Finland population register. The reimbursement finishes if the person dies or stays abroad for >1 year.

All mycobacterium diagnostics requiring cultures are performed in the Tuberculosis Laboratory of the National Institute for Health and Welfare (THL), which notifies the National Infectious Disease Register (NIDR) in the case of a new finding of *Mycobacterium tuberculosis*. Between 1995 and 2006, physicians also had to notify the NIDR about a positive culture, histologically confirmed cases of TB and sputum smear-positive pulmonary TB. As of 2007, the statistics have also included clinically diagnosed cases.

The patients were identified by cross-referencing these two national registers. The Social Insurance Institution of Finland provided data on adult patients with reimbursed DMARDs between 1966 and 2007, and the NIDR provided data on adult patients with a TB notification between 1995 and 2007. The number receiving yearly reimbursement for DMARDs came from the register of the Social Insurance Institution. The TB incidence of the general population came from NIDR data on adult patients with a TB notification.

### Variables and case identification

We accessed individual social security numbers, the date of the TB notification and the hospital where the clinical samples were obtained or where the notification was made. An internal medicine and infectious disease specialist reviewed the original individual medical records. After confirming RD and TB diagnoses, patients with an RD and TB episode were identified as cases. The TB diagnosis was accepted if it was bacteriologically confirmed or, if clinically diagnosed, the case had histological or radiological evidence of TB, or the diagnosis was made differentially [22].

Additional recorded information about patients selected as cases included smoking and drinking history, detailed information on DMARD use, and co-morbidity diagnoses and RD diagnoses, all based on the International Classification of Diseases, 10th revision. If the date of the RD diagnosis was unavailable, the date recorded was when RD was first mentioned. We collected TB exposure status, tuberculin skin test (TST) positivity, radiological findings and details on the TB infection.

The TST was considered positive if the induration was over 5 mm or over 10 mm in Bacillus Calmette–Guérin-vaccinated patients. Thorax X-rays or computed tomography scans were examined if there was suspicion of active intrathoracic TB, suspicion of a previous episode of TB, or no suspicion of TB.

Tuberculosis was considered to be disseminated if present in at least two non-contiguous organs.

Latent TB infection is defined by the World Health Organization as a state of persistent immune response to stimulation by *M. tuberculosis* antigens with no evidence of clinically manifest active TB [23]. No gold standard exists for diagnosing LTBI. During our study period, IFNγ-releasing assays (IGRAs) were not in clinical use in Finland. We considered LTBI likely if the patient was born in a high-incidence country, had a positive TST, had imaging findings that indicated an earlier episode of TB, or had known TB exposure or previously treated TB. The earlier treatment was considered adequate according to the Finnish contemporary guidelines if the patient had received at least two effective anti-TB drugs for ≥12 (1950–1969) or 6 months (1970 and thereafter).

The outcome was assessed 6 months after discontinuation of TB treatment. Treatment was determined to be completed successfully if it lasted at least 6 months, and by absence of failure. We recorded death before, during or within 6 months after TB treatment. If TB was mentioned as a cause of death, death was determined as the outcome.

The coordinating ethics committee of Helsinki and Uusimaa Hospital District and the ethics committee of Carea (Kymenlaakso Social and Health Services) approved the study.

### Statistical analysis

Summary statistics are presented as means or medians, with s.d. or interquartile ranges (IQRs). To compare independent samples for continuous variables, we used Student’s unpaired *t*-test or the Wilcoxon rank sum test, whichever was appropriate. For categorical variables, we used the χ^2^ test.

To investigate incidence trends, we used a Poisson regression model. For the regression coefficient, we calculated an average marginal effect that displays the average change in the annual incidence during the study period. Data handling and statistical analyses were performed with Microsoft Office and R (v.3.4.0, R Foundation for Statistical Computing, Vienna, Austria).

## Results

The register data linkage covering 988 049 patient-years with reimbursed DMARDs yielded 388 individual patients during 1995–2007, and of these, we accessed the medical records for 366 (94.3%). After reviewing their medical records, we confirmed 291 as cases: 66 patients had no RD at the time of TB (diagnosis), but 31 of these received their RD diagnoses after a TB episode. Eight patients had no TB, and in one patient TB was diagnosed in 1994. Of the 291 RD patients accepted who had TB, in 251 (86.3%) TB was confirmed bacteriologically. Of the 40 TB cases diagnosed clinically, 33 (82.5%) were verified histologically, 4 (10%) were treated because of radiological findings and 3 after strong clinical suspicion of TB without another explanation for their condition.

### Patients

The basic data for the 291 patients are shown in Table 1. Of these patients, 196 had RA, and 95 had another RD. The various diagnoses of RD numbered >20, with the largest sub-groups being polymyalgia rheumatica (20%), AS (13.6%) and SLE (10.5%).

**Table 1.** Characteristics of patients

Patient characteristics	All	RA	Other RD	
	(*n* = 291)	(*n* = 196)	(*n* = 95)	
Age, mean (s.d.), range, years	70 (13), 15–93	71 (12), 17–92	68 (15), 15–93	
Sex, *n* (%)				
Male	105 (36)	69 (35)	36 (38)	
Female	186 (64)	127 (65)	59 (62)	
Disease duration, mean (s.d.), years	15 (13)	18 (13)	10 (11)	
Smoking habits, *n* = 187, *n* (%)				
Non-smoker		65 (52)	37 (61)	
Current smoker		27 (22)	15 (24)	
Ex-smoker		33 (26)	9 (15)	
Co-morbidities, *n* = 289, *n* (%)				
Pulmonary disease		57 (29)	22 (23)	
Diabetes		25 (13)	16 (17)	
Kidney disease		20 (10)	8 (8)	
Medication for RD, *n* (%)				
Glucocorticoids		123 (63)	57 (60)	
sDMARDs		123 (63)	40 (42)	*P* < 0.01
TNFi		21 (11)	4 (4)	
Extra-articular RA manifestations, *n* (%)				
Amyloidosis		15 (8)		
Rheumatic pulmonary disease		15 (8)		
Sicca syndrome		8 (4)		
Felty's syndrome		1 (0.5)		
Other extra-articular manifestation^a^		21 (11)		

The age, sex and duration of rheumatic disease of those with any RD who developed active TB in Finland during 1995–2007 are shown. Smoking habits, common co-morbidities and RD medication at the time of diagnosis in RA patients and in patients with RDs other than RA listed separately. The extra-articular disease manifestations of RA patients are listed.

a Vasculitis, cutaneous manifestations, ceratitis, immune thrombocytopenia, Raynaud's phenomenon, lymphangitis.

RD: rheumatic disease; sDMARDs: synthetic DMARDs; TB: tuberculosis; TNFi: TNF inhibitors.

There were 127 seropositive RA patients (64.8% of the RA cases). There were 182 RA patients with co-morbidities (92.9%; mean number of co-morbidities three, IQR two to five). Three patients had received immunosuppressive therapy for pulmonary disease, and one for a renal transplant. Patients described as/known to be heavy users of alcohol numbered 15 (7.7%); eight (4.1%) had an active malignancy. None had HIV infection.

Of all 291 RD patients, 228 (78.4%) had received glucocorticoids or any DMARDs at the time of TB diagnosis, 26 (8.9%) used or had used TNFi, and one patient had previously used an IL-1 receptor antagonist (anakinra).

Of those patients treated with bDMARDs, 19 (73.1%) were screened for LTBI, 11 had at least one known TB exposure, 4 a positive TST, and 4 suspicion of a previous episode of TB on thoracic X-ray. Only one had received LTBI treatment.

Among the RD patients, at least one exposure to TB was reported in 83 (28.5%), and 62 (74.7%) were exposed to TB through active disease in one or more relatives or household contacts. Of those patients aged 15–54 years, seven (21.2%) reported occupational exposures, which is a significantly larger proportion than the nine (3.5%) occupational exposures reported by those ≥ 55 years (*P* < 0.001). No statistically significant difference emerged between the reported number of TB exposures between the oldest patients (≥ 66 years or more) and the younger patients.

### Incidence

**Fig. 1:**
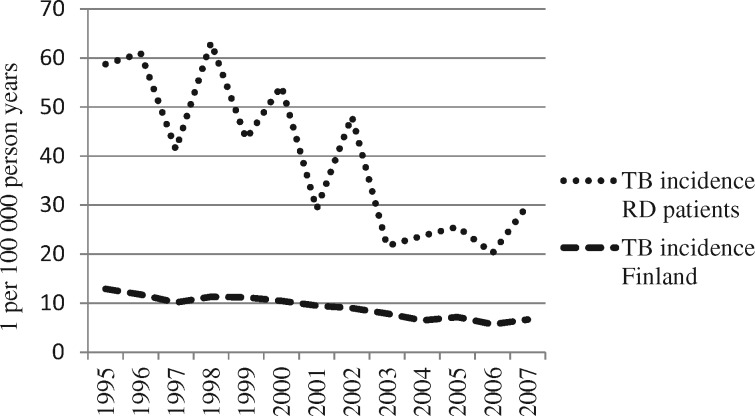
Tuberculosis incidence in Finland, 1995–2007 Annual incidences (one per 100 000 person years) of tuberculosis (TB) in Finland among the general population as reported by the National Infectious Disease Register (NIDR) were significantly lower (*P* < 0.01) than incidences among patients with rheumatic disease (RD). The incidence of both diminished during the study period (*P* < 0.05).

Between 1995 and 2007, the incidence of TB in adult RD patients with reimbursed DMARDs decreased from 58.8 to 30.0 per 100 000 (trend *P* < 0.001, average marginal effect −3.4/100 000 per year, 95% CI −4.4, −2.4; Fig. 1). The incidence was ∼4-fold that of the general population. Table 2 lists the mean numbers of annual cases of TB in the patients whose medical records we accessed.

**Table 2.** Mean yearly tuberculosis cases

**Tuberculosis cases annually, mean (IQR)**	1995–2000	2001–2007	1995–2007
RD	26.83 (22–29)	18.57 (15–20)	22.38 (15–28)
RD with bDMARDs		3.71 (2–6)	
RD without bDMARDs		14.86 (12–15)	20.24 (14–28)
RA	17.50 (14–18)	13.00 (10–13)	15.08 (11–16)
RA with bDMARDs		3.14 (2–5)	
RA without bDMARDs		9.86 (7–9)	13.38 (8–16)

The number of yearly tuberculosis cases with the IQR for time intervals 1995–2000 before bDMARDs, 2001–2007 after bDMARDs came into clinical use, and for the whole study period. Numbers are for all RD patients and for RA patients only.

bDMARDs: biological DMARDs; IQR: interquartile range; RD: rheumatic disease.

### Tuberculosis

**Fig. 2:**
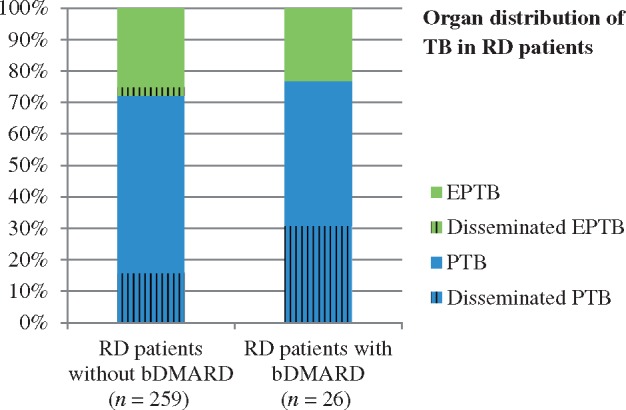
Organ distribution of tuberculosis in rheumatic disease patients (expressed as a percentage) No statistically significant difference appeared in the organ distribution of TB in patients without bDMARDs and in patients taking such drugs. bDMARD: biological DMARD; EPTB: extra-pulmonary tuberculosis; PTB: pulmonary tuberculosis; RD: rheumatic disease; TB: tuberculosis.

The clinical picture of TB was known in 285 (97.9%) cases; pulmonary TB was the most common form of TB among RD patients (72.6%). We found 10 (3.5%) cases of isolated TB pleurisy and found other extra-pulmonary forms of TB in 68 (23.9%) patients. Disseminated TB was present in 56 patients (19.6%). Figure 2 shows the organ distribution of TB for RD patients treated or not treated with bDMARDs.

A history of TB was identified for 36 RA patients. TST was performed in 13 (36.1%) previously treated for TB: 5 (38.5%) were TST negative (3 of these without DMARDs) and 8 (61.5%) were TST positive. Among the previously treated RA patients, only four (11.1%) had received adequate TB treatment. Of all 291 RD patients, reactivation of their LTBI was the reason for infection in 279 cases (95.9%), and among RA patients in 189 (96.4%).

The mean time from symptom onset to TB diagnosis was 16 weeks in all RA patients (median 9 weeks, IQR 4–20 weeks), and for previous and current bDMARD users it was 12 weeks (median 11 weeks, IQR 7–16 weeks). Seven patients lacked any subjective symptoms of TB, and 10 were diagnosed post-mortem.

One hundred and seven (58.5%) RA patients completed their treatment successfully; 58 (31.7%) died.

## Discussion

The annual incidence of TB in patients with RDs was four times as high as in the general population. This result is consistent with an RA cohort study from Spain (an intermediate-incidence country) and the studies from Korea and Sweden [6, 17, 24]. During our study period, the incidence of TB declined by almost 50%. This trend is similar to that in the general population and can be explained by an improved standard of living and by strict TB control during the 20th century: a network of TB hospitals, the TB Act 1948 and the Bacillus Calmette–Guérin vaccine given to all children born in hospitals as a part of the national vaccination programme 1941–2006 [18, 19].

The declining TB incidence in RD patients despite increasing clinical TNFi use reflects the cohort effect of older generations with possible LTBI infection dying, but it is evidence of effective LTBI screening and treatment and of patient selection. Given that TNFi were expensive, these were used first by young and working people.

The main reason for the higher incidence of TB in RD patients is that both RDs and TB are more common in the elderly, but RD patients may have some special vulnerability to TB in addition to the use of CSs, sDMARDs and TNFi; LTBI might even play a role in the onset of RD [25, 26]. Reactivation of LTBI was estimated to be the cause of active TB in almost all cases, although only 30% of the patients had at least one reported exposure to TB. Although the oldest patients were born when pulmonary TB was common, reported exposure rates were similar to those in the younger patients. This is perhaps related to the old stigma of TB, which can make it difficult for older people to mention their past TB exposures to medical staff.

We found no evidence of increasing TB incidence among those with RD. As TB incidence in the population decreases, the number of exposures also decreases. In a low-incidence country, the rate of recently acquired active infections remains low because TB transmission is rare. In a high-incidence country, we would probably see more recently acquired TB infections [27].

Given that new medications help to retain functional capacity, people with RDs may continue working for longer [1, 28, 29]. In future, potential occupational exposure requires attention, especially with regard to treatment of patients with TNFi. People from low-incidence TB countries may also be exposed to TB when they travel to high-incidence areas for work or leisure. Physicians should be aware of the TB risk for immigrants from a high-incidence area.

An increased risk of TB is reported for those in contact with TB, for patients undergoing dialysis, underweight people, patients receiving TNFi, health-care workers, immigrants from countries with a high burden of TB, patients with cancer or diabetes, alcohol abusers, smokers and individuals with radiological findings of fibrotic lesions [23, 30, 31]. These risk factors should help clinicians if they suspect LTBI and active TB.

In some RA patients, previous TB was treated before effective anti-TB drugs were available. Other reasons for earlier inadequate TB treatment include side-effects and resistant strains of *M. tuberculosis*. Negative TST in five previously TB-treated RA patients underlines the fact that TST is not a reliable tool when examining patients with immunosuppressive diseases or medication. Therefore, a history of TB and its treatment should govern treatment decisions in patients with RD.

The most prevalent form of TB in the general population is pulmonary, comprising >80% of cases, whereas disseminated TB is rare (5–10%) [32–35]. RA patients treated with TNFi may more often present with disseminated or extra-pulmonary TB [7, 20, 36]. Our RD patients had a rate of disseminated disease ≤ 19.6%, in agreement with the findings of a Japanese study involving 20 RA patients [37].

Delayed diagnosis and treatment of TB may lead to more severe illness and TB transmission. One systematic review indicated that the median duration of delay from symptom onset to treatment initiation ranged from 60 to 90 days [38]. Our mean time from symptom onset to TB diagnosis was 16 weeks, median 9 weeks. A major problem is that TB has no specific symptoms and can be asymptomatic [37]. Active TB infection cannot be ruled out by normal CRP or ESR. Physicians must be aware of pulmonary TB whenever a cough lasts ≥ 3 weeks or a patient is coughing up blood or sputum; it is not unusual, however, for an unsuspicious post-infectious cough to last for ≤ 8 weeks [39]. The diagnosis of extra-pulmonary TB is even more challenging.

The most important matter to assess is possible TB exposure and predisposing factors (age, TNFi, underlying pulmonary or kidney disease, active cancer, current smoking, heavy drinking and underweight). We identified only five RA patients without any predisposition to TB; three of them were born in Finland before 1950, and one had travelled in a high-incidence country before developing the disease.

The fatality rate of RA patients with TB was high when compared with the 3–13% case fatality rate reported by the World Health Organization as European TB treatment outcome data 1996–2005. This may be associated with frailty and with polypharmacy issues that complicate the treatment of RA and co-morbidities.

Owing to our heterogeneous RD group, we focused on the clinical information for patients with RA, a fact which might limit our study. Our study period might be also too short to show the effect of bDMARDs on TB rates, but the incidence trend was still declining. Clinics may vary in notification rates for clinically diagnosed TB cases. Unfortunately, our analysis has demanded considerable time, but these data still offer clinically relevant information on the risk and manifestation of TB among patients with RD.

### Conclusion

The incidence of TB among RD patients was approximately four times as high as that for latent TB of the general population and has declined between 1995 and 2007.

At the time of RD diagnosis, TB exposure and factors predisposing to TB require close evaluation, and re-evaluation is advisable when immunosuppression increases, upon initiation of TNFi therapy, or in the case of new exposure. Systematic testing and treatment of LTBI in populations at risk is crucial in the prevention of active TB [32].

Although the local incidence of TB may be low, overall TB should be kept in mind if a patient with a rheumatic disease presents with otherwise unexplained symptoms or with radiological findings supporting TB.
